# Assessment of Biochemical Bone Turnover Markers in Polish Healthy Children and Adolescents

**DOI:** 10.34763/jmotherandchild.20242801.d-23-00105

**Published:** 2024-04-19

**Authors:** Joanna Gajewska, Magdalena Chełchowska, Grażyna Rowicka, Witold Klemarczyk, Ewa Głąb-Jabłońska, Jadwiga Ambroszkiewicz

**Affiliations:** Department of Screening Tests and Metabolic Diagnostics, Institute of Mother and Child, Warsaw 01-211, Kasprzaka 17a, Poland; Department of Nutrition, Institute of Mother and Child, Warsaw 01-211, Kasprzaka 17a Poland

**Keywords:** osteocalcin, bone alkaline phosphatase, C-terminal telopeptide of type I collagen, children, adolescents, age intervals

## Abstract

**Background:**

Assessing bone turnover in paediatric populations is crucial for understanding the physiological changes occurring during skeletal development and identifying potential abnormalities. The objective of this study was to assess osteocalcin (OC), bone alkaline phosphatase (BALP), and C-terminal telopeptide of type I collagen (CTX-I) levels reflecting bone formation and resorption for age and sex in Polish healthy children and adolescents.

**Materials and Methods:**

A total of 355 healthy normal-weight children and adolescents (46.5% girls) aged 1–18 years old were recruited. Total body less head (TBLH) and spine L1-L4 were used in children to assess bone mineral density (BMD) by dual-energy X-ray absorptiometry (DXA). Bone marker concentrations were determined by immunoenzymatic methods.

**Results:**

Bone marker levels in girls and boys started with higher values in the first year of life and subsequently decreased until reaching a nadir during the prepubertal period. The pubertal peak values of bone markers were reached at 11–13 years old in boys and at 9–11 years old in girls. After puberty, the adolescents showed a gradual decline in bone marker concentrations to the values observed in adults. We found positive correlations between OC level and TBLH-BMD (r = 0.329, p = 0.002), TBLH-BMD Z-score (r = 0.245, p = 0.023), and L1-L4 BMD (r = 0.280, p = 0.009) in the prepubertal group.

**Conclusions:**

We showed serum levels of bone turnover markers—BALP, OC, and CTX-I—in relation to age and sex in healthy Polish children and adolescents. The age intervals of these markers for girls and boys aged 1–18 years old may be clinically useful in the assessment of bone metabolism in individuals with skeletal disorders.

## 1. Introduction

Skeletal health is of paramount importance during childhood and adolescence, as it directly affects growth, development, and overall quality of life. Optimal bone development and maintenance require a proper balance between bone formation and resorption [1]. The process of bone remodelling, which involves the continuous removal of old bone tissue and the subsequent formation of new bone, is pivotal for maintaining skeletal integrity, strength, and mineralization. Imbalances in bone metabolism can lead to growth retardation and a range of skeletal disorders, including osteoporosis and fractures [2].

Assessing bone turnover in paediatric populations is crucial for understanding the physiological changes occurring during skeletal development and identifying potential abnormalities. Traditionally, bone mineral density (BMD) measurements using dual-energy X-ray absorptiometry (DXA) have been widely used to evaluate bone health in children and adolescents [3]. However, BMD alone does not provide a comprehensive understanding of bone turnover dynamics.

In recent years, biochemical markers have emerged as valuable tools for assessing bone metabolism and turnover [4]. These markers—measured in blood or urine samples—provide insights into the balance between bone formation and resorption processes. Among the various bone turnover markers, osteocalcin (OC), bone alkaline phosphatase (BALP), and C-terminal telopeptide of type I collagen (CTX-I) have gained significant attention due to their association with specific aspects of bone remodelling [5].

OC, a non-collagenous protein synthesized by osteoblasts, plays a crucial role in bone mineralization and is considered a marker of the bone formation process [6]. Studies have suggested that OC levels are influenced by factors such as age, sex, puberty, hormonal, and nutritional status. Similarly, bone alkaline phosphatase, an enzyme primarily synthesized by osteoblasts, is involved in bone matrix mineralization [7]. BALP levels have been shown to correlate with bone formation rates and exhibit variations during different stages of growth and development. On the other hand, CTX-I is a degradation product of collagen released during the bone resorption process [8]. As bone resorption occurs, CTX-I is released into the bloodstream, making it a reliable marker of the bone resorption activity of osteoclasts. CTX-I levels have been found to increase during pubertal growth spurts and decrease following interventions that suppress bone resorption, such as bisphosphonate therapy [9].

Although numerous studies have explored bone turnover markers in various populations, limited research has focused specifically on healthy children and adolescents [10,11,12,13,14]. Establishing reference ranges for these markers in healthy paediatric populations is crucial for understanding normal bone metabolism during growth and development. Moreover, reference ranges can be useful to improve clinical practices by aiding in the early detection of bone health disorders and diseases in children and adolescents. Therefore, the primary objective of this study was to assess the levels of OC, BALP, and CTX-I reflecting bone formation and resorption for each age interval, separately for boys and girls, in Polish healthy children and adolescents.

## 2. Materials and methods

### 2.1. Patients

A total of 355 healthy children and adolescents (165 girls, 190 boys) aged 1–18 years old with a BMI Z-score <−1 + 1> were recruited to the study group. Densitometric examination was performed in the group of 88 children aged 5 to 10 years old. On the basis of data from the history and medical examination, these children were: (a) without either acute or chronic disorders, among them obesity; (b) without bone fractures in the period up to one year before the study; (c) not taking any medications that could affect their growth, pubertal development, nutritional or dietary status; (d) whose parents signed the informed consent form. All of the participants were Caucasian. Pubertal stage was determined according to the Tanner scale by the physician during the medical appointment.

Physical examinations, including body height and weight measurements, were performed in prepubertal children. The Body Mass Index (BMI) was calculated as body weight divided by height squared (kg/m^2^). The BMI of each individual was converted to a standard BMI Z-score for the child’s age and sex using Polish reference tables [15]. Densitometry examination was performed in the studied children in two projections: total body and spine L1-L4. Total body less head (TBLH) were used for performing bone mineral content (BMC) and BMD by DXA, (Lunar Prodigy, General Electric Healthcare, Madison, WI, paediatric software 9.30.044). All subjects were measured on the same machine. The measurements were performed using standard positioning techniques.

Written informed consent was obtained from the parents of all the examined children. The study was conducted between September 2012 and December 2018 in accordance with the Helsinki Declaration for Human Research, and the study protocol was approved (protocol code no. 03/09) by the Ethics Committee of the Institute of Mother and Child in Warsaw, Poland.

### 2.2. Biochemical methods

Venous blood samples were collected between 8:00 and 10:00 a.m. after an overnight fast and centrifuged at 1000 × g for 10 min at 4°C. Serum specimens were stored at −70°C prior to assay (no longer than for six months). Biochemical parameters were determined by immunoenzymatic methods. BALP activity was estimated using the BAP EIA kit from Quidel (Athens, USA) with a within-assay variability of less than 5.8% and a between-assay variability of less than 7.6%. OC and CTX-I concentrations were measured with the N-MID OC ELISA kit and serum CrossLaps ELISA kit (IDS, Bolton, UK), respectively. The intra- and inter-assay coefficients of variation were less than 2.2% and 5.1% for OC, and 3.0% and 10.9% for CTX-I. The detection limit was 0.7 U/L for BALP, 0.5 ng/mL for OC, and 0.02 ng/mL for CTX-I.

### 2.3. Statistical analyses

Statistical analysis was performed using Statistica 6.0 (StatSoft Inc.) software. The results are presented as medians and interquartile ranges (25^th^–75^th^ percentiles) for non-normally distributed variables. The Kolmogorov–Smirnov test and graphical inspections of data were used to evaluate distribution for normality. Differences in the anthropometric characteristics and biochemical parameters of healthy girls and boys were assessed using the non-parametric Mann–Whitney *U* test. Spearman correlations between the anthropometric and biochemical parameters were calculated. Differences were regarded as statistically significant at *p* < 0.05.

## 3. Results

The general distribution of bone markers in 355 subjects (165 girls and 190 boys) are shown in Table 1. The group of girls and group of boys were of similar age and did not differ in terms of OC and CTX-I levels. However, BALP activity was higher by about 10% in boys than girls (*p* = 0.048).

**Table 1.** Bone markers in the studied population of children and adolescents.

**Variable**	**Girls**	**Boys**	**Total**	*P* ^*^
Participants, n (%)	165 (46.5)	190 (53.5)	355 (100)	0.189
Age (years)	8.5 (5.5–12.0)	8.4 (5.5–13.0)	8.4 (5.5–12.3)	0.981
BALP (U/L)	106.6 (84.6–128.4)	115.4 (92.0–136.5)	112.1 (87.9–132.5)	**0.048**
OC (ng/mL)	84.0 (52.6–114.4)	80.9 (55.5–116.9)	83.2 (52.6–114.4)	0.518
CTX-I (ng/mL)	1.72 (1.12.–2.22)	1.67 (1.22.–2.09)	1.70 (1.19–2.12)	0.805

Medians and interquartile ranges (25th–75th percentiles),

* p-values between girl and boy groups. BALP–bone alkaline phosphatase, OC–osteocalcin, and CTX-I–C-terminal telopeptide of type I collagen.

**Figure 1.**
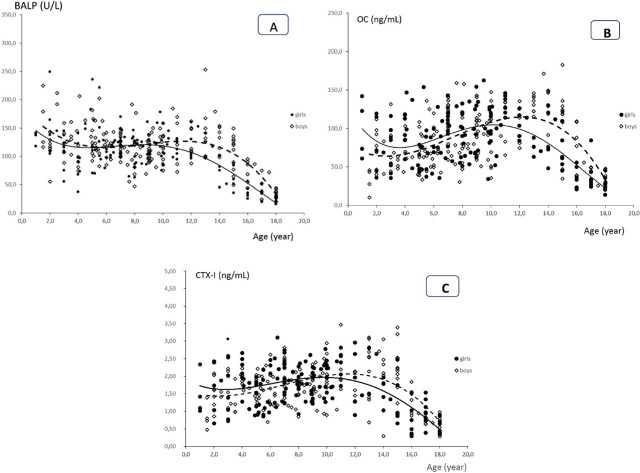
BALP **(A)**, OC **(B)**, and CTX-I **(C)** trend curves (polynomial trendlines: 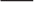 girls, boys -----) with individual data points for girls and boys aged 1–18 years old.

BALP, CTX-I, and OC reference curves for children and adolescents aged 1–18 years old are shown in the Fig. 1 (A,B,C). In general, all the studied bone markers in girls and boys started with higher values in the first year of life and subsequently decreased until reaching a nadir during the prepubertal period, earlier in girls than boys. The pubertal peak values of bone markers were reached at 9–11 years old in girls and at 11–13 years old in boys. In girls, after the age of 11 years old, a gradual decrease in marker values was observed, until the lowest values were reached at the age of 18 years old. In boys, after the age of 13 years old, continuous decreases in the values of these parameters until age 18 were also observed.

Characteristics of the study population with age and sex stratification are presented in Table 2. Reference ranges of bone markers in children and adolescents were defined as medians, between 25th and 75th percentiles of serum concentrations. From our results, the observed bone markers varied significantly in adolescents, with sex differences. Median values of BALP at ages 14–15.9 years old and 16–18 years old were higher in boys than girls (*p* < 0.001; *p* = 0.022, respectively). Median values of OC at ages 12–13.9 years old, 14–15.9 years old, and 16–18 years old were higher in boys than in girls (*p* = 0.019; *p* = 0.010; *p* = 0.018, respectively). The median values of CTX-I at ages 14–15.9 were higher in boys than in girls (*p* = 0.004).

**Table 2.** Bone markers in children and adolescents aged 1–18 years old stratified by sex and age.

**Parameter/years**	**Girls (n=165)**			**Boys (n=190)**			***p***
	
	**n**	**Median**	**Range**	**n**	**Median**	**Range**	
**BALP (U/L)**							
1.0–2.9	10	139.5	122.8–143.9	14	126.7	116.3–170.2	0.861
3.0–4.9	20	112.8	86.7–138.3	19	125.5	100.7–142.4	0.227
5.0–7.9	43	117.9	102.5–132.1	53	118.7	103.6–138.8	0.945
8.0–9.9	32	118.8	98.4–128.0	33	109.7	94.8–125.7	0.568
10.0–11.9	16	109.4	97.2–129.9	14	118.6	88.3–152.7	0.618
12.0–13.9	10	120.7	101.0–138.8	18	129.1	101.8–190.1	0.598
14.0–15.9	12	67.2	49.5–84.4	18	120.9	99.3–134.4	**<0.001**
16.0–18.0	22	34.2	20.7–39.7	21	44.1	30.8–71.6	**0.022**
**OC (ng/mL)**							
1.0–2.9	10	72.0	62.6–118.0	14	57.8	41.3–81.8	0.160
3.0–4.9	20	89.7	53.7–113.5	19	62.5	52.4–77.1	0.092
5.0–7.9	43	77.3	58.0–105.7	53	77.4	62.8–116.6	0.749
8.0–9.9	32	95.6	69.9–126.1	33	92.2	74.0–110.0	0.436
10.0–11.9	16	114.8	95.7–127.1	14	101.0	66.2–132.3	0.406
12.0–13.9	10	88.8	77.9–111.2	18	122.5	96.0–135.8	**0.019**
14.0–15.9	12	73.5	59.0–110.0	18	122.0	110.5–134.0	**0.010**
16.0–18.0	22	30.4	23.2–41.3	21	38.8	30.3–49.5	**0.018**
**CTX-I (ng/mL)**							
1.0–2.9	10	1.58	1.05–2.22	14	1.40	0.90–1.59	0.266
3.0–4.9	20	1.94	1.23–2.37	19	1.66	1.21–2.02	0.369
5.0–7.9	43	1.80	1.25–2.17	53	1.85	1.33–2.14	0.651
8.0–9.9	32	1.80	1.43–2.25	33	1.67	1.49–1.98	0.240
10.0–11.9	16	2.23	1.72–2.43	14	1.91	1.79–2.19	0.383
12.0–13.9	10	1.74	1.47–2.64	18	2.13	1.41–2.66	0.905
14.0–15.9	12	1.37	0.95–1.79	18	2.19	1.96–2.50	**0.004**
16.0–18.0	22	0.72	0.41–0.97	21	0.86	0.64–0.95	0.375

Medians and interquartile ranges (25th–75th percentiles),

* *p*-values between girl and boy age groups. BALP–bone alkaline phosphatase, OC–osteocalcin, and CTX-I–C-terminal telopeptide of type I collagen.

In the group of girls, we found negative correlations between age and BALP activity (*r* = −0.475, *p* < 0.001), OC (*r* = −0.202, *p* = 0.01), and CTX-I levels (*r* = −0.248, *p* = 0.001). In the group of boys, a negative correlation was found between age and BALP activity (*r* = −0.348, *p* < 0.001), but a positive correlation between age and OC level (*r* = 0.155, *p* = 0.033). In the whole study group of children and adolescents, we observed negative correlations between age and BALP activity (*r* = −0.402, *p* < 0.001), and CTX-I level (*r* = −0.118, *p* = 0.026).

Positive correlations were found in the whole study group as well as in the girl and boy groups separately between CTX-I and OC levels (*r* = 0.664, *p* < 0.001; *r* = 0.687, *p* < 0.001; *r* = 0.645, *p* < 0.001, respectively), and between CTX-I level and BALP activity (*r* = 0.207, *p* < 0.001; *r* = 0.256, *p* = 0.001; *r* = 0.173, *p* = 0.017, respectively). Positive correlations were also found between OC level and BALP activity in the whole study group and the girls subgroup (*r* = 0.170, *p* = 0.001; *r* = 0.219, *p* = 0.005, respectively).

Characteristics of the prepubertal children (45 girls, 43 boys) group are presented in Table 3. Age, anthropometric parameters, and bone markers did not show any differences between the two sex groups. However, in the whole group of prepubertal children, we found positive correlations between BMI Z-score and TBLH-BMD (*r* = 0.282, *p* = 0.008), TBLH-BMD Z-score (*r* = 0.366, *p* < 0.001), TBLH-BMC (*r* = 0.266, *p* = 0.012), L1-L4 BMD (*r* = 0.248, *p* = 0.021), and L1-L4 BMD Z-score (*r* = 0.265, *p* = 0.014) values (Table 4). In addition, we observed positive correlations between OC level and TBLH-BMD (*r* = 0.329, *p* = 0.002), TBLH-BMD Z-score (*r* = 0.245, *p* = 0.023), TBLH-BMC (*r* = 0.329, *p* = 0.002), and L1-L4 BMD (*r* = 0.280, *p* = 0.009) values in this group.

**Table 3.** Serum bone marker concentrations and anthropometric parameters in prepubertal healthy children aged 5–10 years old.

**Variable**	**Girls n=45**	**Boys n=43**	**Total n=88**	*p* ^*^
Age (years)	8.2 (6.8–9.1)	7.5 (5.6–9.0)	8.0 (6.0–9.1)	0.081
Weight (kg)	27.0 (21.3–29.4)	23.9 (20.3–29.9)	25.1 (20.4–29.7)	0.361
Height (cm)	128.5 (119.0–133.8)	125.6 (117.8–132.6)	126.4 (118.6–133.5)	0.278
BMI (kg/m^2^)	15.5 (14.5–17.0)	15.6 (14.6–16.6)	15.5 (14.6–16.8)	0.910
BMI Z–score	−0.36 (−0.70–0.24)	−0.37 (−0.75–0.04)	−0.37 (−0.74–0.18)	0.841
TBLH-BMD (g/cm2)	0.686 (0.642–0.737)	0.644 (0.622–0.720)	0.672 (0.627–0.730)	0.246
TBLH-BMD Z-score	−0.40 (−0.70–0.20)	−0.10 (−0.60–0.40)	−0.30 (−0.63–0.20)	0.101
TBLH-BMC (g)	600.6 (452.8–764.5)	526.2 (419.4–751.3)	582.8 (436.2–760.3)	0.258
L1-L4 BMD (g/cm^2^)	0.666 (0.631–0.731)	0.624 (0.569–0.707)	0.659 (0.595–0724)	0.057
L1-L4 BMD Z-score	−0.40 (−0.80–0.30)	−0.40 (−1.20–0.20)	−0.4 (−1.00–0.20)	0.296
BALP (U/L)	119.8 (100.3–131.3)	116.7 (102.0–130.5)	117.9 (100.3–131.4)	0.799
OC (ng/mL)	81.2 (63.8–102.7)	75.2 (63.5–94.4)	77.5 (63.7–100.5)	0.450
CTX-I (ng/mL)	1.78 (1.37–2.16)	1.66 (1.44–2.00)	1.73 (1.39–2.02)	0.419

Medians and interquartile ranges (25th–75th percentiles),

* p-values between girl and boy groups.

BMI–body mass index, TBLH–total body less head, BMD–bone mineral density, BMC–bone mineral content, BALP–bone alkaline phosphatase, OC–osteocalcin, and CTX-I–C-terminal telopeptide of type I collagen.

**Table 4.** Relations between BMI Z-score, bone markers and densitometric parameters in prepubertal healthy children aged 5–10 years old.

**Parameters**	**TBLH-BMD r (p)**	**TBLH-BMD Z-score r (p)**	**TBLH-BMC r (p)**	**L1-L4 BMD r (p)**	**L1-L4 BMD Z-score r (p)**
BMI Z-score	**0.282 (0.008)**	**0.366 (<0.001)**	**0.266 (0.012)**	**0.248 (0.021)**	**0.265 (0.014)**
BALP	−0.049 (0.651)	−0.092 (0.398)	−0.032 (0.769)	−0.019 (0.861)	0.006 (0.95)
OC	**0.329 (0.002)**	**0.245 (0.023)**	**0.329 (0.002)**	**0.280 (0.009)**	0.192 (0.077)
CTX-I	0.078 (0.476)	0.048 (0.661)	0.101 (0.356)	0.097 (0.374)	0.051 (0.051)

BMI–body mass index, TBLH–total body less head, BMD–bone mineral density, BMC–bone mineral content, BALP–bone alkaline phosphatase, OC–osteocalcin, and CTX-I–C-terminal telopeptide of type I collagen.

## 4. Discussion

The application and interpretation of the results regarding bone turnover markers is more complicated in children than in adults. Children and adolescents have elevated circulating concentrations of biochemical bone markers, reflecting high growth velocity and rapid bone turnover [8].

In adults, osteoblasts and osteoclasts participate only in bone remodelling, while in children they play a role in both the bone remodelling process and bone growth, including longitudinal growth and increases in bone circumference and thickness [16]. The use of bone markers in children and adolescents is increasing, for example—in Paget’s disease, treatment-induced bone diseases such as glucocorticoid-induced osteoporosis and other chronic bone disorders including those accompanying autoimmune diseases in children, such as inflammatory bowel disease, food allergies, or chronic kidney diseases [17,18,19,20,21]. Therefore, there is a need to establish reference values of bone turnover markers for developmental age for boys and girls separately.

Some authors described the use of bone marker values for children and adolescents from the age of a few months to 17–18 years old or only for adolescence [22,23,24,25,26]. Age and sex are important variables that affect bone formation and resorption marker concentrations in childhood and adolescence [27–28]. Usually, two peaks of bone growth were observed with the highest bone marker levels during the developmental period. The first peak was in infants and children during the first four years of life [10,29] and the second was in children during the pubertal period [14,23]. Bone marker concentrations were slightly lower and rather stable between these two peaks. After the pubertal period, girls and boys showed a gradual decline in bone marker concentrations to the values observed in young adults [26].

In our study, BALP values were similar for both sexes in the group of small children, but the peak in puberty was slightly higher in boys and occurred later than in girls. During puberty, BALP showed a peak at 9–11 years of age in girls, while this was 2–3 years later in boys. These results are in agreement with data obtained in German and multi-ethnic populations by other authors [30,31]. Our study also showed elevated OC—second marker of bone formation—during infancy but more in girls than boys. However, the peak of OC during puberty was higher and occurred 2–3 years later in boys than in girls.

The literature data on serum OC concentrations are ambiguous. Rauchenzauner et al. [32] found rather stable results in the Austrian population. Other authors observed, similar to us, increased OC concentrations during infancy which decreased before reaching a peak during early puberty in Central European and Danish populations [14,33]. In terms of the bone resorption marker CTX-I, we observed a peak during the pubertal period only and it was slightly higher and occurred about two years later in boys than in girls. Other authors also did not show a peak of CTX-I in early infancy, but found that it was only slightly increasing to reach a peak of this marker in early puberty in Canadian and European populations [29,34].

In the presented study, we analysed bone markers and anthropometric parameters in prepubertal healthy children. We used TBLH as a scanning site when assessing BMD according to The International Society for Clinical Densitometry (ISCD) recommendations [35]. We observed similar values of height, weight, BMI and BMI Z-score, TBLH-BMD, TBLH-BMC, L1-L4 BMD, and L1-L4 BMD Z-score as well as bone markers in girls and boys. We found positive relations between BMI Z-score and TBLH-BMD, TBLH-BMD Z-score, TBLH-BMC, L1-L4 BMD, and L1-L4 BMD Z-score in prepubertal children.

The influence of obesity on bone turnover and bone quality in children and adolescents remains controversial [36]. Some researchers observed a significantly greater whole-body bone area and BMC for age and for height in obese children and adolescents [37], while others described a lower bone mass and bone area in obese children compared with normal weight subjects [38]. Our earlier and present studies in prepubertal children confirm the relations between greater values of BMI Z-score and greater bone mass (BMC, BMD, and BMD Z-score) [39].

We also found positive relations between OC and TBLH-BMD, TBLH Z-score, TBLH-BMC, and spine L1-L4 BMD in prepubertal children. However, we did not observe any relations between BALP, CTX-I levels, and DXA measurement values in this study group. Paldanius et al. [24] found no associations between BMD with OC values in healthy Finnish children and adolescents in an older age group (7 to 19 years old) than the group examined in this study. Zurcher et al. [40] and Monjardino et al. [41] also found no associations of CTX-I with DXA measurements in Swiss and Portuguese populations of children and adolescents.

According to Jurimae [42], the interpretation of bone metabolism in children and adolescents should not be based on one bone turnover marker but several markers reflecting different steps in the bone formation and resorption processes. In our study, we showed the concentrations of three markers concerning bone turnover stratified by age and sex in healthy children and adolescents. The use of bone markers requires compliance with a number of pre-analytical and analytical procedures.

Various reagent kits of bone turnover markers are available and may not give identical results for these markers. OC is an unstable protein, hence there is a problem with selecting appropriate kits for determining this marker in serum. According to Rosenquist et al. [43], immunoassays targeting the NH2-terminal middle fragment (N-middle OC) are preferred for routine testing, because the C-terminal OC sequence is the least stable. BALP was defined as the marker of choice in haemodialyzed patients [44]. This marker is almost not impacted by renal function, fasting, or circadian rhythm [45]. However, there are reported cases of transient hyperphosphatasaemia in infants under five years old [46]. In these cases, BALP activity was transiently increased but returned to normal values after a few weeks without any symptoms. CTX-I, analysed in our study using the CrossLaps kit, was influenced by fasting, circadian rhythm, and renal failure [45]. To reduce preanalytical biochemical bone marker variance, blood samples should be collected after an overnight fast and within the same four-hour time frame for all participants. Thus, the effect of circadian rhythm and food intake on tested marker levels will be limited [45].

The ISCD indicates that early identification and treatment of children at risk of fractures is necessary due to the possibility of restoring bone strength to growing children [47]. Therefore, the use of additional diagnostic and treatment monitoring methods, such as biochemical markers of bone turnover, would be useful to better identify patients affected by paediatric osteoporosis or those at highest risk of other skeletal disorders. In particular, reference values of bone turnover markers could be useful in monitoring bisphosphonate treatment in children with Juvenile Paget’s disease, osteogenesis imperfecta, and osteoporosis [48,49,50]. These markers may be helpful in estimating an effective inhibition of osteoclast function with reduction of bone resorption during therapy.

In conclusion, we showed serum levels of bone turnover markers BALP, OC, and CTX-I in relation to age and sex in healthy Polish children and adolescents. In this study, associations between OC and DXA measurements in prepubertal children were also found. The age intervals of the studied markers for healthy girls and boys aged 1–18 years old may be clinically useful in the assessment of bone metabolism in children and adolescents with primary skeletal disorders and those accompanying other diseases.
